# Changes in Sexuality and Sexual Dysfunction over Time in the First Two Years after Treatment of Head and Neck Cancer

**DOI:** 10.3390/cancers15194755

**Published:** 2023-09-27

**Authors:** Margot A. Stone, Birgit I. Lissenberg-Witte, Remco de Bree, Jose A. Hardillo, Femke Lamers, Johannes A. Langendijk, C. René Leemans, Robert P. Takes, Femke Jansen, Irma M. Verdonck-de Leeuw

**Affiliations:** 1 Department Otolaryngology-Head and Neck Surgery, Amsterdam UMC, Vrije Universiteit Amsterdam, 1081 HV Amsterdam, The Netherlands; m.a.stone@student.vu.nl (M.A.S.); cr.leemans@amsterdamumc.nl (C.R.L.); f.jansen1@amsterdamumc.nl (F.J.); 2 Cancer Center Amsterdam, Treatment and Quality of Life, 1081 HV Amsterdam, The Netherlands; b.lissenberg@amsterdamumc.nl; 3 Department of Epidemiology and Data Science, Amsterdam UMC, Vrije Universiteit Amsterdam, 1081 HV Amsterdam, The Netherlands; 4 Department of Head and Neck Surgical Oncology, University Medical Center Utrecht, 3584 CS Utrecht, The Netherlands; r.debree@umcutrecht.nl; 5 Department of Otorhinolaryngology, Erasmus MC, University Medical Center, 3000 CA Rotterdam, The Netherlands; j.hardillo@erasmusmc.nl; 6 Department of Psychiatry, Amsterdam UMC, 1081 HV Amsterdam, The Netherlands; f.lamers@amsterdamumc.nl; 7 Amsterdam Public Health Research Institute, 1081 HV Amsterdam, The Netherlands; 8 Department of Radiation Oncology, University Medical Center Groningen, 9713 GZ Groningen, The Netherlands; j.a.langendijk@umcg.nl; 9 Department of Otorhinolaryngology-Head and Neck Surgery, Radboud University Medical Center, 6500 HB Nijmegen, The Netherlands; robert.takes@radboudumc.nl; 10 Department of Clinical, Neuro- and Developmental Psychology, Vrije Universiteit Amsterdam, 1081 HV Amsterdam, The Netherlands

**Keywords:** head and neck cancer, sexuality, sexual dysfunction, surgery, radiotherapy, chemotherapy

## Abstract

**Simple Summary:**

Many head and neck cancer patients report sexual problems. A deterioration in sexuality and sexual dysfunction from baseline to 3 months after treatment was observed especially in patients treated with chemoradiation. This effect seems to differ between men and women. Men reported change in erectile function, orgasm, satisfaction with intercourse, and overall satisfaction, while women reported change in desire, arousal, and orgasm. These findings are helpful to improve information on sexuality and sexual care in head and neck cancer patients.

**Abstract:**

The aim of this study was to investigate changes in sexuality and sexual dysfunction in head and neck cancer (HNC) patients in the first two years after treatment, in relation to the type of treatment. Data were used of 588 HNC patients participating in the prospective NETherlands Quality of life and Biomedical Cohort Study (NET-QUBIC) from diagnosis to 3, 6, 12 and 24 months after treatment. Primary outcome measures were the International Index of Erectile Function (IIEF) and the Female Sexual Function Index (FSFI). The total scores of the IIEF and FSFI were dichotomized into sexual (dys)function. In men, type of treatment was significantly associated with change in erectile function, orgasm, satisfaction with intercourse, and overall satisfaction. In women, type of treatment was significantly associated with change in desire, arousal, and orgasm. There were significant differences between treatment groups in change in dysfunctional sexuality. A deterioration in sexuality and sexual dysfunction from baseline to 3 months after treatment was observed especially in patients treated with chemoradiation. Changes in sexuality and sexual dysfunction in HNC patients were related to treatment, with an acute negative effect of chemoradiation. This effect on the various domains of sexuality seems to differ between men and women.

## 1. Introduction

Per year, more than half a million people are diagnosed with head and neck cancer (HNC) worldwide [1]. HNC treatment affects, among other aspects of quality of life, sexuality and intimacy [2]. Prevalence rates of sexual dysfunction vary between 24% and 100% [2,3,4,5,6,7,8]. Surgery can cause disfigurement, scarring, and loss of tissue, impairing functions and appearances in the head and neck area, such as speaking and kissing. Radiotherapy is known to cause skin problems and dry mouth as a result of radiation damage in tissues surrounding the targeted tumor in the head and neck area. As a consequence of its systemic toxicity, chemotherapy can influence the central nervous system and hormone production. However, current knowledge on the impact of HNC treatment on sexuality and sexual dysfunction among HNC patients is based on studies that were mostly cross-sectional and/or used the EORTC QLQ-HN35 questionnaire with only two sexuality items, and/or had small sample sizes, and/or included all patients while some of them were not sexually active [2,3,4,5,6,7,8,9]. More in-depth information from sexual-specific questionnaires is lacking on the main sexual domains: sexual desire, arousal, orgasm, satisfaction, pain, lubrication, and erectile function. Also, we need to know more about the course of (dysfunctional) sexuality over time from diagnosis up to two years after treatment, in relation to type of treatment, both in the total patient population as well as those who are sexually active.

The aim of this study was to estimate changes over time in sexuality and sexual dysfunction among HNC patients in the first two years after diagnosis and treatment, in relation to type of treatment. We hypothesize that changes in sexuality and sexual dysfunction over time are different depending on treatment type. The results of this study are relevant to guide future research on sexuality in HNC patients and to develop personalized sexual care in HNC patients who suffer from sexual dysfunction after treatment.

## 2. Materials and Methods

### 2.1. Patients and Procedures

Data were used from The Netherlands Quality of Life and Biomedical Cohort Study in head and neck cancer (NET-QUBIC) [10]. NET-QUBIC is a longitudinal observational cohort study comprising clinical records and data deriving from patient reported outcome measures (PROMs), fieldwork assessment (interviews and functional tests), and collection of biosamples. Patients were recruited in five out of the eight Dutch HNC centers between 2014 and 2018. Details of the key components of NET-QUBIC have been published previously: the study population (including retention and attrition), the electronic case report form (eCRF), the outcome assessment protocol, biobanking protocol, data management (collection and storage), and data and sample dissemination procedures [10].

Inclusion criteria were newly diagnosed squamous cell carcinomas in the head and neck (oral cavity, oropharynx, hypopharynx, larynx, unknown primary; all stages); age  >  18 years; treatment with curative intent; surgery, radiotherapy, chemotherapy, or a combination of these treatments; able to write, read, and speak Dutch. Exclusion criteria were other tumors in the head and neck (e.g., lymphoma, skin malignancies, thyroid cancer); unable to understand the questions or test instructions; severe psychiatric co-morbidities (e.g., Korsakoff’s syndrome, severe dementia). In the current study on sexuality, the primary outcomes measures were the PROMs Female Sexual Function Index (FSFI) and the International Index of Erectile Function (IIEF). HNC survivors were included in the current study if they filled out the FSFI or IIEF on at least one time point.

The study protocol of NET-QUBIC was approved by the Institutional Review Board of Amsterdam UMC (location VUmc) (2013.301(A2018.307)-NL45051.029.13). All participants provided written informed consent.

### 2.2. Sample Size Calculation

Sample size calculation was based on the research question of the entire NET-QUBIC study, i.e., to describe the course of health-related quality of life over time, with a difference over 60 months of 4 points change on the global quality of life scale between categories of relevant variables, using a residual standard deviation (SD) of 10 points within categories, and using an α of 0.05 and a power (β) of 0.80. For the dependency of the 5 repeated assessment points, we assumed an intraclass correlation coefficient of 0.50. This resulted in a total sample size of 462.

### 2.3. Outcome Measures

Detailed descriptions of all outcome measures including references can be found in the NET-QUBIC data catalogue (https://researchers.kubusproject.nl/data-catalogue (accessed on 22 September 2023)). A short description is provided below.

Demographic (age, sex) and clinical data (tumor site (oral cavity, oropharynx, hypopharynx, larynx, unknown primary); human papilloma virus (HPV) presence in oropharyngeal tumors (yes/no); clinical disease stage (categorized into stage I, II, III, or IV); treatment type (categorized into surgery, radiotherapy, surgery and radiotherapy, and chemotherapy with or without radiotherapy and or surgery), World Health Organization (WHO) performance status, and comorbidity (the Adult Comorbidity Evaluation-27 Index (none/mild/moderate/severe) were obtained from clinical records.

The IIEF and FSFI were completed at baseline (approximately 2 weeks after diagnosis, before treatment) and 3, 6, 12, and 24 months after treatment. All items of the IIEF and FSFI refer to the past four weeks, and higher total and subscale scores indicate better sexuality [11,12].

The IIEF is a 15-item questionnaire that represents five domains of male sexuality: sexual desire (2 items, range 1–10), erectile function (6 items, range 0–30), orgasm (2 items, range 0–10), intercourse satisfaction (3 items, range 0–15), and overall satisfaction (2 items, range 1–10). The total score of the IIEF ranges from 0 to 75 points [11]. In the current study, patients who scored >0 on the intercourse satisfaction subscale (indicating having had sexual intercourse in the past 4 weeks) at any time point were considered to be sexually active.

The FSFI is a 19-item questionnaire that represents six domains of female sexuality: desire (2 items; range 1.2–6), arousal (4 items; range 0–6), lubrication (4 items, range 0–6), orgasm (3 items, range 0–6), satisfaction (3 items, range 0.4–6), and pain (3 items), range 0–6). The total score of the FSFI ranges from 1.6 to 36 points [12]. In the current study, patients who had a total FSFI score ≥ 6 (indicating having had sexual intercourse in the past 4 weeks) at any time point were considered to be sexually active.

Sexual dysfunction was based on the validated total FSFI cut-off score of <25.5 [13] and the total IIEF cut-off score of <53 [14].

### 2.4. Statistical Analysis

Descriptive statistics were generated to describe the study population. Chi square tests and independent *t*-test were used to compare descriptive statistics between the included and excluded patients.

To investigate changes over time in the scores of the (sub)scales of the IIEF and the FSFI, overall linear mixed model (LMM) analyses (with compound symmetry as covariance structure) were performed with fixed effects for time (categorical) and random effects for subject. Secondly, LMM analyses were performed investigating the association between changes over time and treatment type (time × treatment type) These second analyses were adjusted for age, tumor site and stage (as fixed factors). Sensitivity analyses were performed repeating these LMM analyses, excluding HNC survivors who were not considered sexually active.

To investigate changes in sexual dysfunction over time among HNC survivors who were sexually active, generalized estimating equations (GEE) analyses were performed investigating the association between change over time and treatment type. Reference category in the GEE analysis was the treatment modality with the lowest percentage of dysfunctional sexuality at baseline. The GEE analyses were adjusted for age, sex, tumor site and stage.

In all analyses a *p*-value < 0.05 was considered statistically significant. All analyses were performed using the IBM Statistical package for the Social Sciences (SPSS) version 28 (IBM Corp., Armonk, NY, USA).

## 3. Results

### 3.1. Study Population

**Figure 1:**
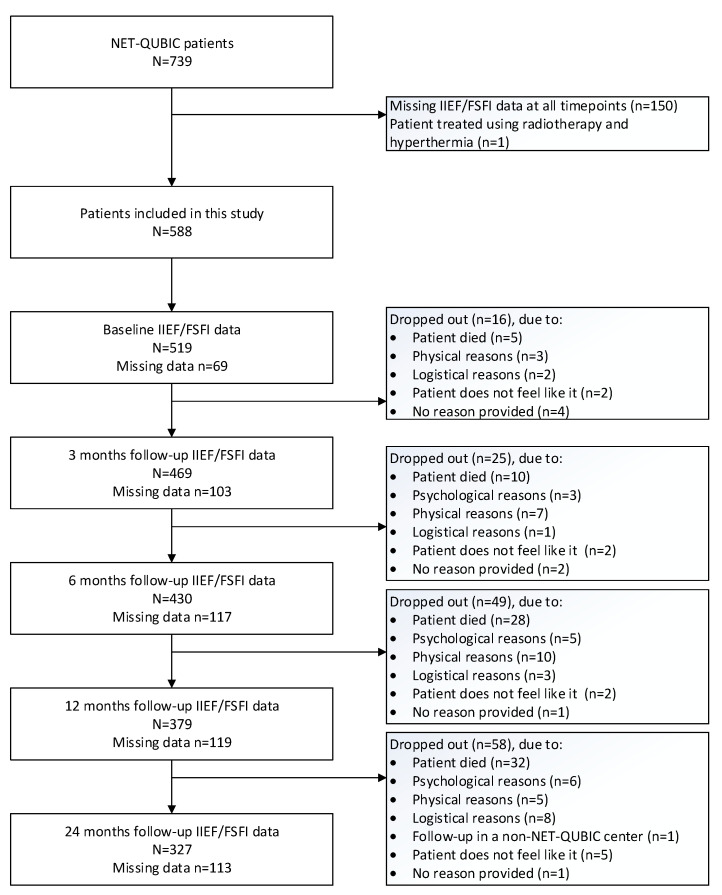
Flow diagram of the study population.

The flow diagram of the study is presented in Figure 1. Of the 739 patients participating in NET-QUBIC, 588 were included in the current study (79.5%) (Figure 1).

Of the 588 included HNC survivors, 323 survivors (55%) were sexually active on at least one time point. An overview of the characteristics of the study populations is provided in Table 1.

The current study population (N = 588) differed significantly from the excluded NET-QUBIC patients (N = 151) regarding living situation, HPV status, tumor stage, WHO performance, and comorbidity. Excluded patients more often lived alone, more often had an HPV-negative oropharynx tumor, more often had a larger tumor stage, and more often had comorbidity (Table 1).

### 3.2. Changes over Time in Sexuality

Results of the overall LMM analyses are shown in Table 2.

In men, there was a significant change over time on the IIEF overall satisfaction scale, with a deterioration from baseline to three months after treatment and improved scores from six months onwards. In women, there were no significant changes over time on any of the FSFI scales. Among sexually active men and women, there were no significant changes over time.

Results of the LMM analyses on differences between treatment groups in change over time in sexuality are presented in Table 3.

In men (IIEF), type of treatment was significantly associated with change over time in erectile function, satisfaction with sexual intercourse, and overall satisfaction. When adjusted for age, tumor site and stage, type of treatment was significantly associated with change over time in erectile function, orgasm, satisfaction with intercourse, and overall satisfaction. Among sexually active men, the unadjusted and adjusted LMM analyses showed that type of treatment was significantly associated with change over time in erectile function, satisfaction with intercourse, and overall satisfaction. In women (FSFI), in the unadjusted and adjusted analyses, type of treatment was significantly associated with change over time in desire and arousal. Among sexually active women, in the unadjusted and adjusted LMM analyses, type of treatment was significantly associated with change over time in desire, arousal, and orgasm.

### 3.3. Changes over Time in Sexual Dysfunction among Sexually Active HNC Patients

Results of the GEE analyses are shown in Table 4.

**Figure 2:**
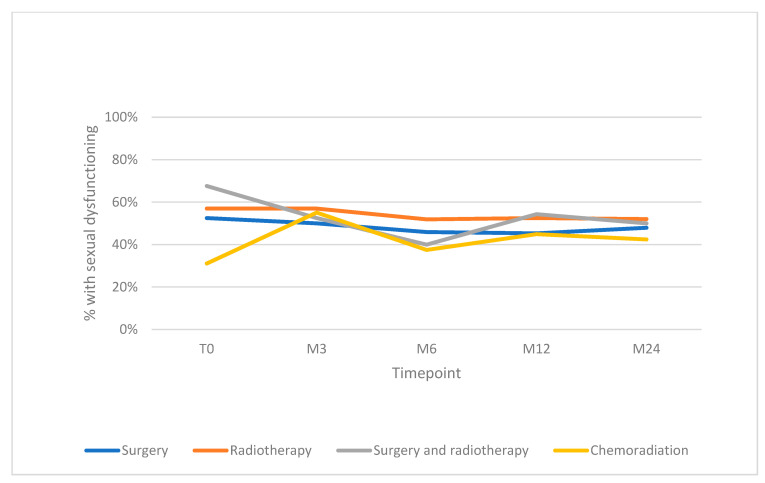
The course of dysfunctional sexuality in HNC survivors from baseline to 3, 6, 12, and 24 months after treatment, in relation to type of treatment.

The group treated by chemoradiation with or without surgery had the lowest percentage of sexual dysfunction at baseline (i.e., 31% versus 52–68% in the other treatment groups) and was selected as the reference group (Table 4). The unadjusted GEE analysis showed a significant association between type of treatment and change over time in sexual dysfunction (*p* = 0.038), as did the GEE analyses adjusted for age, sex, tumor site and stage (*p* = 0.019) (Table 4). A visualization of the change of sexual dysfunction (in percentage) over time in relation to treatment type is shown in Figure 2.

In the group treated with chemoradiation with or without surgery, the percentage of patients with sexual dysfunction increased after treatment to 3 months after treatment up to the level of the other groups; the percentages in these groups remained stable (single treatment by surgery or radiotherapy) or decreased (surgery and radiotherapy) at 3 months after treatment compared to baseline. From 3 to 6 months after treatment, the percentage of patients with sexual dysfunction decreased in all groups but the most in the groups treated by multimodal treatment (surgery and radiotherapy, and chemoradiation with or without surgery). From 6 to 12 months there was an increase in the percentage of patients with sexual dysfunction in the multimodal treatment groups. From 12–24 months, percentages of patients with sexual dysfunction remained more or less stable over time in all groups.

## 4. Discussion

In this study, we investigated changes over time in sexuality and sexual dysfunction from baseline to two years after treatment among HNC patients, in relation to the type of HNC treatment. In men, type of treatment was significantly associated with change over time in erectile function, orgasm, satisfaction with intercourse, and overall satisfaction. In women, type of treatment was significantly associated with change over time in desire and arousal. Among sexually active HNC patients, type of treatment was significantly associated with change over time in erectile function, satisfaction with intercourse and overall satisfaction (men) and in desire, arousal, and orgasm (women). Among sexually active HNC patients, there were also significant differences between the treatment groups in change over time in sexual dysfunction. An acute negative effect (deterioration in sexuality and sexual dysfunction from baseline to three-month follow-up, and improvement afterwards) was especially observed in patients treated with chemoradiation (either with or without surgery).

Before treatment, the group of patients to be treated with chemoradiation with or without surgery had the lowest percentage of dysfunctional sexuality. In men, this group of patients also scored the highest (best) on all IIEF subscales before treatment. In women, the group with the highest (best) scores on the FSFI scales were women to be treated with surgery alone, most often followed by women to be treated with chemoradiation. An explanation for the finding that patients to be treated with chemoradiation are among those having the best sexuality at baseline may be that in clinical practice the choice of treatment is made deliberately and partly dependent on a patient’s health condition and age. Patients who are offered chemotherapy adjuvant to surgery and radiotherapy are often those with a younger age, HPV-positive tumor and those in reasonably good health. Nevertheless, after adjustment for age, sex, tumor site and stage, the association remained significant.

It was striking that patients treated with chemoradiation (with or without surgery) showed significantly more deterioration in sexuality and increase in dysfunctional sexuality from baseline to 3 months after treatment, compared to the other groups, in both men and women. This may have been caused by the finding that this group had the lowest percentage of dysfunctional sexuality and scores could deteriorate more, but it also indicates that chemotherapy as such or in combination with radiotherapy and/or surgery has an acute negative effect on sexuality. This negative effect confirms a previous prospective study among HNC patients treated with (chemo)radiation. Melissant et al. [7] found that, among 354 HNC patients, before start of treatment, 37% of patients reported having less sexuality, which increased to 60% at 6-week follow-up, and returned to baseline level from 12-month follow-up onwards. Patients who received chemoradiation had worse sexuality than patients treated with radiotherapy only [7]. A recent study by Humbert et al. [9] investigated sexual health among 241 HNC survivors five years after diagnosis. They asked participants whether their sexual health had altered compared to the time of diagnosis. Sexual health had decreased according to 79% of the survivors. The decrease in sexual health was related to radiotherapy and/or chemotherapy, as well as to fatigue, impaired physical health, and higher disease stage.

The negative effect of chemoradiation on sexuality may be explained by an effect on testosterone levels. In a previous pilot study among 40 HNC patients (all treatment modalities) of whom 10–25% had testosterone insufficiency, testosterone was significantly associated with sexual problems before as well as 6 months after treatment [15]. In men, testosterone was significantly associated with orgasm before treatment and sexual desire at 6 months after treatment. In women, testosterone was not significantly associated with sexual problems before treatment, but with sexuality and intimacy and sexual satisfaction at 6 months after treatment [15]. In the current study, we also observed differences between men and women in the way their sexuality was affected by HNC treatment. In men, significant changes over time were found in erectile function, orgasm, satisfaction with intercourse and overall satisfaction (but not in sexual desire), while in women, the effect of treatment was observed in sexual desire, arousal, and orgasm (but not in lubrication, pain, or satisfaction). These findings indicate that more research is needed to explain the effect of treatment on testosterone and sexuality.

Further research might focus on the female androgen insufficiency syndrome (FAIS) (women) or late onset hypogonadism (men). In women, low testosterone and impaired sexual function in combination with poor well-being, dysphoric mood, and possibly also fatigue, vasomotor instability, vaginal dryness, decreased muscle strength, poor memory, and bone loss are criteria of FAIS [16] In men, late onset hypogonadism is a clinical syndrome that includes testosterone insufficiency in combination with symptoms such as reduced sexual desire, erectile dysfunction, and decreased spontaneous erections, as well as low mood, decreased motivation, and fatigue decreasing vigorous activity, with difficulty walking >1 km, and decreased bending [17]. Previous research among HNC patients found that less sexuality was associated with older age, trouble with social contact, weight loss, and constipation [7]. It has also been suggested that the negative effects of cancer treatment on sexuality are associated with physical symptoms, such as pain, xerostomia, poorer social–emotional functioning, physical barriers (e.g., feeding tube) and appearance changes [2]. Neijenhuijs et al. [18] carried out an explorative study on symptom clusters, using machine learning techniques on a PROM data set from 1032 cancer patients (all types of cancer). Data on 26 symptoms (physical and psychosocial) were analyzed. There appeared to be a difference in the co-occurrence of symptoms dependent on symptom severity. With respect to sexuality, in patients with high-risk scores, sexuality and psychological complaints clustered together in a “general sickness” cluster as well as in a “physical symptoms and consequences” cluster. In patients with moderate-to-high risk scores, sexuality and psychological complaints were not clustered together. More research will provide more insight into possible syndromes of symptoms related to testosterone and sexuality in HNC patients.

Strengths of this study are the large group size at inclusion, including both men and women, the prospective study design, and the use of questionnaires specifically focusing on sexuality. Limitations of this study include relatively smaller group sizes at follow-up (especially among women) and the binary categorization of gender which might exclude people who do not consider themselves as males or females. Furthermore, the population from NET-QUBIC differs slightly from the Dutch population in The Netherlands Cancer Registry (NCR) regarding sex, age, tumor site and treatment modality, affecting the generalizability of our results [10]. Also, there were differences between the study population in the current study and the entire NET-QUBIC study population regarding HPV status, tumor stage, WHO performance, and comorbidity. However, we suspect that many HNC patients who were not sexually active did not fill out the FSFI and IIEF and were therefore excluded. The results in this study among the sexually active HNC patients may therefore be considered as representative. Lastly, although the FSFI and IIEF are commonly used questionnaires to assess sexual function, they do have some limitations. The focus is primarily on heterosexual intercourse and penetrative sexual activity, which may not capture the sexual experience of patients with other sexual orientations or those with non-penetrative sexual activities. Also factors such as body image concerns and relationship dynamics are not addressed by the FSFI or IEFF.

Despite these limitations, from a clinical perspective the results of this study are relevant. Many HNC patients have an unmet need for sexual care [19]. According to guideline and recommendations [20,21], it is recommended to discuss sexual health and dysfunction resulting from cancer or its treatment with the patient. Based on the current results, clinicians can better inform patients about a possible negative effect of chemoradiation on their sexuality: they can inform women that this negative effect is especially to be expected in desire, arousal and orgasm and they can inform men that this can be expected in erectile function, orgasm, satisfaction with intercourse, and overall satisfaction.

## 5. Conclusions

Changes in sexuality and dysfunctional sexuality in HNC patients are related to treatment, with an acute negative effect of chemoradiation. This effect on the various domains of sexuality seems to differ between men and women. Clinicians can use this information to develop tailored sexual care interventions for HNC patients.

## Figures and Tables

**Table 1:** Overview of the study population at baseline.

Characteristics	Included HNC Patients N = 588	Included HNC Patients Who Were Sexually Active N = 323	Excluded HNC Patients (No Data on Sexuality) N = 151	Difference between Included (N = 588) and Excluded Patients *p* Value
Mean age in years (SD)	63.3 ± 9.5	61.9 ± 9.8	63.2 ± 10.5	0.97
Gender				0.65
- Male	439 (74.7%)	249 (77.1%)	110 (72.8%)	
- Female	149 (25.3%)	74 (22.9%)	41 (27.2%)	
Living situation *				<0.001
- Alone	121 (22.1%)	41 (13.4%)	42 (41.2%)	
- Living with partner with or without children	397 (72.6%)	249 (81.4%)	54 (52.9%)	
- Other	29 (5.3%)	16 (5.2%)	6 (5.9%)	
Tumor location				0.16
- Oral cavity	165 (28.1%)	94 (29.1%)	34 (22.5%)	
- Oropharynx	208 (35.4%)	113 (35.0%)	54 (35.8%)	
- Hypopharynx	36 (6.1%)	12 (3.7%)	16 (10.6%)	
- Larynx	160 (27.2%)	92 (28.5%)	45 (29.8%)	
- Unknown primary	19 (3.2%)	12 (3.7%)	2 (1.3%)	
HPV status (oropharynx only) *				0.004
- Positive	112 (61.5%)	69 (71.1%)	18 (38.3%)	
- Negative	70 (38.5%)	28 (28.9%)	29 (61.7%)	
TNM stage				0.018
- I	141 (24.0%)	86 (26.6%)	22 (14.6%)	
- II	110 (18.7%)	58 (18.0%)	22 (14.6%)	
- III	99 (16.8%)	52 (16.1%)	28 (18.5%)	
- IV	238 (40.5%)	127 (39.3%)	79 (52.3%)	
Treatment				0.24
- Surgery	129 (21.9%)	77 (23.9%)	23 (15.4%)	
- Radiotherapy	191 (32.5%)	99 (30.7%)	50 (33.6%)	
- Surgery and radiotherapy	86 (14.6%)	47 (14.6%)	20 (13.4%)	
- Chemoradiation with or without surgery	182 (31.0%)	99 (30.7%)	56 (37.6%)	
WHO performance				0.002
- Able to carry out normal activity	414 (70.4%)	236 (73.1%)	93 (61.6%)	
- Restricted in physical activity	150 (25.5%)	79 (24.5%)	41 (27.2%)	
- Ambulatory	24 (4.1%)	8 (2.5%)	17 (11.3%)	
Comorbidity *				0.007
- None	178 (31.7%)	115 (37.5%)	26 (19.0%)	
- Mild	212 (37.7%)	116 (37.8%)	52 (38.0%)	
- Moderate	113 (20.1%)	53 (17.3%)	42 (30.7%)	
- Severe	59 (10.5%)	23 (7.5%)	17 (12.4%)	

Abbreviations: N number; SD standard deviation; HPV Human Papilloma Virus; TNM tumor site and size (T), N regional lymph node involvement (N), distant metastatic spread (M); WHO World Health Organization; * numbers do not add up due to missing data.

**Table 2:** Results of the overall linear mixed model analyses on the course of the subscales of the IIEF (men) and FSFI (women) over time.

	All Patients						Sexually Active Patients
	Baseline	3 Months	6 Months	12 Months	24 Months		
IIEF	N, mean (SD)	N, mean (SD)	N, mean (SD)	N, mean (SD)	N, mean (SD)	*p*-value ^1^	*p*-value ^2^
Desire	391 5.3 (2.3)	356 5.3 (2.3)	334 5.5 (2.3)	293 5.5 (2.4)	251 5.6 (2.2)	0.34	0.23
Erectile function	367 13.7 (11.2)	335 13.4 (10.9)	312 14.1 (11.3)	278 14.3 (11.2)	237 14.5 (11.1)	0.92	0.64
Orgasm	391 4.8 (4.5)	354 4.8 (4.5)	330 5.0 (4.6)	294 5.1 (4.5)	244 5.3 (4.5)	0.93	0.77
Intercourse satisfaction	389 4.2 (5.3)	355 4.1 (5.3)	333 4.6 (5.4)	293 4.6 (5.5)	247 4.7 (5.4)	0.67	0.78
Overall satisfaction	357 6.4 (2.6)	326 6.1 (2.6)	305 6.5 (2.6)	269 6.5 (2.6)	225 6.6 (2.5)	0.01	0.033
FSFI							
Desire	128 2.2 (1.2)	113 2.3 (1.2)	96 2.2 (1.2)	85 2.3 (1.3)	76 2.2 (1.2)	0.88	0.99
Arousal	124 1.9 (2.2)	110 1.8 (2.1)	95 1.8 (2.2)	81 2.0 (2.2)	72 2.1 (2.3)	0.75	0.65
Lubrication	128 1.9 (2.5)	110 2.0 (2.4)	95 1.8 (2.5)	82 2.2 (2.6)	71 2.0 (2.5)	0.83	0.90
Orgasm	124 1.9 (2.4)	109 1.9 (2.4)	95 1.8 (2.4)	82 2.1 (2.5)	70 1.9 (2.5)	0.74	0.74
Satisfaction	111 3.1 (2.0)	89 3.0 (1.9)	84 2.8 (2.0)	69 3.1 (1.9)	64 2.8 (1.8)	0.34	0.13
Pain	117 2.0 (2.6)	106 2.0 (2.6)	88 2.0 (2.7)	76 1.9 (2.6)	66 1.7 (2.5)	0.61	0.38

Abbreviations: N number; SD standard deviation; IIEF International Index of Erectile Function; FSFI Female Sexual Function Index; ^1^ LMM analyses among all patients; ^2^ LMM analyses among sexually active patients.

**Table 3:** Associations between type of treatment and changes in IIEF (men) and FSFI (women) scales over time.

	**All Men**							**Sexually Active Men**	
	**Baseline**	**3 Months**	**6 Months**	**12 Months**	**24 Months**				
IIEF	N, mean (SD)	N, mean (SD)	N, mean (SD)	N, mean (SD)	N, mean (SD)	*p*-value ^2^	*p*-value ^3^	*p*-value ^4^	*p*-value ^5^
Desire						0.26	0.25	0.063	0.062
Surgery	N = 77, 5.3 (2.2)	N = 79. 5.5 (2.2)	N = 75, 5.5 (2.1)	N = 68, 5.6 (2.3)	N = 59, 5.5 (2.0)				
Radiotherapy	N = 136, 4.9 (2.3)	N = 116, 5.3 (2.4)	N = 115, 5.3 (2.4)	N = 92, 5.4 (2.4)	N = 83, 5.3 (2.3)				
Surgery and radiotherapy	N = 53, 5.1 (2.3)	N = 52, 5.1 (2.1)	N = 42, 5.6 (2.2)	N = 41, 5.5 (2.4)	N = 34, 5.6 (2.4)				
Chemoradiation ^1^	N = 125, 5.7 (2.4)	N = 109, 5.3 (2.5)	N = 102, 5.8 (2.4)	N = 92, 5.6 (2.5)	N = 75, 5.9 (2.3)				
Erectile function						0.041	0.038	0.043	0.034
Surgery	N = 70, 13.6 (10.8)	N = 75, 14.9 (10.9)	N = 68, 15.1 (10.8)	N = 63, 14.5 (11.1)	N = 57, 13.8 (10.5)				
Radiotherapy	N = 130, 12.5 (10.9)	N = 110, 12.0 (10.5)	N = 109, 12.1 (11.0) (4.5)	N = 88, 12.5 (11.1)	N = 76, 12.5 (11.0)				
Surgery and radiotherapy	N = 44, 11.7 (10.8)	N = 48, 12.4 (10.8)	N = 39, 15.1 (10.4)	N = 38, 14.8 (10.8)	N = 33, 14.1 (11.5)				
Chemoradiation ^1^	N = 123, 15.8 (11.7)	N = 102, 14.2 (11.2)	N = 96, 15.4 (12.0)	N = 89, 15.7 (11.4)	N = 71, 17.3 (11.3)				
Orgasm						0.0504	0.048	0.108	0.095
Surgery	N = 77, 4.8 (4.4)	N = 79, 5.6 (4.4)	N = 74, 5.2 (4.5)	N = 69, 5.3 (4.5)	N = 59, 5.3 (4.4)				
Radiotherapy	N = 141, 4.3 (4.5)	N = 118, 4.1 (4.4)	N = 113, 4.5 (4.5)	N = 92, 4.6 (4.5)	N = 78, 4.5 (4.5)				
Surgery and radiotherapy	N = 50, 3.9 (4.4)	N = 50, 4.3 (4.5)	N = 41, 5.4 (4.5)	N = 40, 5.7 (4.4)	N = 34, 5.4 (4.6)				
Chemoradiation^1^	N = 123, 5.7 (4.6)	N = 107, 5.0 (4.6)	N = 102, 5.3 (4.7)	N = 93, 5.3 (4.6)	M = 73, 6.0 (4.5)				
Intercourse satisfaction						0.010	0.010	0.009	0.009
Surgery	N = 75, 3.5 (4.9)	N = 81, 4.4 (5.2)	N = 74, 4.8 (5.1)	N = 68, 4.5 (5.2)	N = 58, 4.3 (5.0)				
Radiotherapy	N = 141, 3.6 (5.1)3.6 (5, )	N = 117, 4.0 (5.2)	N = 115, 4.1 (5.4)	N = 93, 3.8 (5.6)	N = 82, 4.3 (5.5)				
Surgery and radiotherapy	N = 49, 3.4 (4.5)	N = 50, 3.7 (4.9)	N = 43, 4.4 (5.3)	N = 39, 4.8 (5.0)	N = 33, 4.4 (5.3)				
Chemoradiation ^1^	N = 124, 5.4 (5.9)5.4 (5.9)	N = 107, 4.3 (5.5)	N = 101, 5.1 (5.8)	N = 93, 5.2 (5.9)	N = 74, 5.6 (5.7)				
Overall satisfaction						0.001	0.001	0.013	0.012
Surgery	N = 72, 6.1 (2.4)	N = 78, 5.9 (2.7)	N = 69, 6.3 (2.5)	N = 63, 6.1 (2.6)	N = 54, 6.3 (2.3)				
Radiotherapy	N = 122, 6.2 (2.8)	N = 104, 6.5 (2.5)	N = 103, 6.5 (2.7)	N = 82, 6.5 (2.8)	N = 74, 6.6 (2.8)				
Surgery and radiotherapy	N = 48, 5.7 (2.5)	N = 46, 5.8 (2.4)	N = 39, 6.6 (2.3)	N = 37, 6.5 (2.7)	N = 30, 6.5 (2.6)				
Chemoradiation ^1^	N = 115, 6.9 (2.4)	N = 98, 5.9 (2.7)	N = 94, 6.5 (2.8)	N = 87, 6.9 (2.6)	N = 67, 6.9 (2.4)				
	**All Women**							**Sexually Active Women**	
	**Baseline**	**3 Months**	**6 Months**	**12 Months**	**24 Months**				
FSFI	N, mean (SD)	N, mean (SD)	N, mean (SD)	N, mean (SD)	N, mean (SD)	*p*-value ^2^	*p*-value ^3^	*p*-value ^4^	*p*-value ^5^
Desire						0.003	0.004	0.031	0.026
Surgery	N = 30, 2.5 (1.3)	N = 30, 2.8 (1.3)	N = 27, 2.5 (1.4)	N = 21, 2.5 (1.5)	N = 19, 2.4 (1.5)				
Radiotherapy	N = 35, 1.9 (0.9)	N = 27, 2.1 (1.1)	N = 23, 2.1 (1.2)	N = 22, 2.1 (1.1)	N = 19, 1.9 (0.9)				
Surgery and radiotherapy	N = 22, 1.9 (1.0)	N = 22, 2.2 (1.0)	N = 17, 2.4 (1.3)	N = 18, 2.3 (1.2)	N = 15, 2.4 (1.2)				
Chemoradiation ^1^	N = 41, 2.3 (1.4)	N = 34, 1.9 (1.1)	N = 29, 1.9 (1.1)	N = 24, 2.2 (1.4)	N = 23, 2.2 (1.2)				
Arousal						0.024	0.033	0.020	0.020
Surgery	N = 29, 2.8 (2.4)	N = 29, 2.7 (2.4)	N = 26, 2.4 (2.5)	N = 20, 2.4 (2.6)	N = 19, 2.6 (2.7)				
Radiotherapy	N = 35, 1.4 (1.8)	N = 27, 1.7 (1.9)	N = 23, 1.7 (2.1)	N = 22, 1.8 (2.1)	N = 18, 1.6 (2.0)				
Surgery and radiotherapy	N = 22, 1.4 (2.0)	N = 21, 1.9 (2.1)	N = 16, 2.5 (2.3)	N = 16, 2.3 (2.1)	N = 14, 2.5 (2.3)				
Chemoradiation ^1^	N = 38, 1.9 (2.3)	N = 33, 1.2 (1.8)	N = 30, 1.1 (1.8)	N = 23, 1.7 (2.1)	N = 21, 1.7 (2.1)				
Lubrication						0.06	0.09	0.06	0.07
Surgery	N = 29, 2.7 (2.7)	N = 30, 2.7 (2.6)	N = 27, 2.4 (2.6)	N = 21, 2.6 (2.8)	N = 19, 2.6 (2.9)				
Radiotherapy	N = 38, 1.4 (2.3)	N = 26, 2.0 (2.4)	N = 23, 1.9 (2.6)	N = 21, 2.1 (2.7)	N = 18, 1.4 (2.1)				
Surgery and radiotherapy	N = 22, 1.4 (2.2)	N = 21, 2.0 (2.5)	N = 15, 2.5 (2.7)	N = 16, 2.5 (2.5)	N = 13, 2.6 (2.8)				
Chemoradiation ^1^	N = 39, 2.0 (2.6)	N = 33, 1.3 (2.2)	N = 30, 1.0 (2.0)	N = 24, 1.7 (2.3)	N = 21, 1.6 (2.3)				
Orgasm						0.053	0.08	0.041	0.040
Surgery	N = 27 3.3 (2.6)	N = 29, 2.7 (2.6)	N = 27, 2.5 (2.6)	N = 22, 2.6 (2.8)	N = 19, 2.5 (2.8)				
Radiotherapy	N = 37, 1.3 (2.1)	N = 26, 1.6 (2.1)	N = 23, 1.8 (2.4)	N = 21, 1.8 (2.4)	N = 18, 1.6 (2.3)				
Surgery and radiotherapy	N = 22, 1.3 (2.0)	N = 21, 2.2 (2.6)	N = 15, 2.1 (2.6)	N = 15, 2.7 (2.6)	N = 13, 2.3 (2.6)				
Chemoradiation ^1^	N = 38, 2.0 (2.5)	N = 33, 1.2 (2.1)	N = 30, 0.9 (2.1)	N = 24, 1.6 (2.2)	N = 20, 1.5 (2.4)				
Satisfaction						0.09	0.10	0.17	0.15
Surgery	N = 27, 3.7 (2.1)	N = 24, 3.7 (2.0)	N = 24, 3.3 (2.1)	N = 16, 3.6 (2.2)	N = 15, 3.1 (2.1)				
Radiotherapy	N = 29, 2.7 (1.7)	N = 23, 2.9 (2.0)	N = 20, 2.8 (2.0)	N = 18, 2.7 (1.8)	N = 16, 2.5 (1.5)				
Surgery and radiotherapy	N = 21, 2.9 (2.1)	N = 19, 2.9 (2.0)	N = 14, 3.1 (1.9)	N = 14, 3.5 (1.7)	N = 13, 3.0 (2.0)				
Chemoradiation ^1^	N = 34, 3.2 (2.1)	N = 23, 2.5 (1.7)	N = 26, 2.2 (1.7)	N = 21, 2.7 (1.7)	N = 20, 2.7 (1.7)				
Pain						0.13	0.14	0.13	0.11
Surgery	N = 25, 3.0 (2.6)	N = 27, 2.9 (2.7)	N = 24, 3.0 (2.9)	N = 19, 2.5 (2.8)	N = 16, 2.6 (2.8)				
Radiotherapy	N = 34, 1.6 (2.6)	N = 26, 2.3 (2.8)	N = 21, 2.3 (3.0)	N = 20, 1.9 (2.8)	N = 17, 1.3 (2.3)				
Surgery and radiotherapy	N = 23, 1.7 (2.5)	N = 21, 2.1 (2.7)	N = 14, 2.3 (2.8)	N = 15, 2.9 (2.8)	N = 12, 2.4 (3.0)				
Chemoradiation ^1^	N = 35, 1.9 (2.7)	N = 32, 1.0 (2.2)	N = 29, 0.8 (2.1)	N = 22, 0.8 (1.8)	N = 21, 1.0 (2.0)				

^1^ Chemoradiation alone or combined with surgery. ^2^ LMM analyses on the association between type of treatment and the course of the domains over time among all patients. ^3^ LMM analyses on the association between type of treatment and the course of the domains over time among all patients, adjusted for age, tumor site and stage. ^4^ LMM analyses on the association between type of treatment and the course of the domains over time among sexually active patients. ^5^ LMM analyses on the association between type of treatment and the course of the domains over time among sexually active patients, adjusted for age, tumor site and stage. Abbreviations: LMM = linear mixed models, IIEF = International Index of Erectile Function, FSFI = Female Sexual Function Index.

**Table 4:** Results of GEE-analysis on the course of dysfunctional sexuality over time per treatment group among sexually active patients.

	Unadjusted (*p* = 0.038)		Adjusted (*p* = 0.019) *	
	OR	95% CI	OR	95% CI
3 months				
Surgery	0.33	0.16–0.69	0.29	0.13–0.67
Radiotherapy	0.37	0.19–0.72	0.32	0.15–0.67
Surgery and radiotherapy	0.20	0.08–0.45	0.15	0.06–0.38
Chemoradiation ^1^	Reference			
6 months				
Surgery	0.58	0.27–1.23	0.61	0.26–1.44
Radiotherapy	0.61	0.30–1.27	0.52	0.23–1.18
Surgery and radiotherapy	0.24	0.10–0.61	0.22	0.08–0.63
Chemoradiation ^1^	Reference		Reference	
12 months				
Surgery	0.42	0.19–0.91	0.45	0.18–1.12
Radiotherapy	0.46	0.22–0.96	0.36	0.16–0.82
Surgery and radiotherapy	0.32	0.13–0.77	0.26	0.10–0.70
Chemoradiation ^1^	Reference		Reference	
24 months				
Surgery	0.51	0.22–1.21	0.72	0.27–1.93
Radiotherapy	0.50	0.22–1.16	0.49	0.20–1.24
Surgery and radiotherapy	0.29	0.12–0.75	0.30	0.11–0.84
Chemoradiation ^1^	Reference		Reference	

^1^ Chemoradiation alone or combined with surgery. * Adjusted for age, sex, tumor site, and stage. Abbreviations: OR = odds ratio, CI = confidence interval.

## Data Availability

The data presented in this study are available on request from the corresponding author. The data are not publicly available due to privacy reasons.
